# The ties that bind: an integrative framework of physician-hospital alignment

**DOI:** 10.1186/1472-6963-11-36

**Published:** 2011-02-15

**Authors:** Jeroen Trybou, Paul Gemmel, Lieven Annemans

**Affiliations:** 1 Department of Public Health, Ghent University, Belgium; 2 Department of Management, Innovation and Entrepreneurship, Ghent University, Belgium

## Abstract

**Background:**

Alignment between physicians and hospitals is of major importance to the health care sector. Two distinct approaches to align the medical staff with the hospital have characterized previous research. The first approach, economic integration, is rooted in the economic literature, in which alignment is realized by financial means. The second approach, noneconomic integration, represents a sociological perspective emphasizing the cooperative nature of their relationship.

**Discussion:**

Empirical studies and management theory (agency theory and social exchange theory) are used to increase holistic understanding of physician hospital alignment. On the one hand, noneconomic integration is identified as a means to realize a cooperative relationship. On the other hand, economic integration is studied as a way to align financial incentives. The framework is developed around two key antecedent factors which play an important role in aligning the medical staff. First, provider financial risk bearing is identified as a driving force towards closer integration. Second, organizational trust is believed to be important in explaining the causal relation between noneconomic and economic integration.

**Summary:**

Hospital financial risk bearing creates a greater need for closer cooperation with the medical staff and alignment of financial incentives. Noneconomic integration lies at the very basis of alignment. It contributes directly to alignment through the norm of reciprocity and indirectly by building trust with the medical staff, laying the foundation for alignment of financial incentives.

## Background

The relationship between the hospital and its medical staff is an important area of academic research and a main concern of hospital executives, given the impact on quality of provided care [1], hospitals' financial success [2] and cost-effective healthcare delivery [3]. Internationally, hospitals have evolved from a physician workshop to accountable organizations, charged with the development of internal organizations where quality and cost effectiveness go hand in hand [4]. Consequently, cooperation and alignment between hospitals and their physicians has become paramount to enhance hospital performance. However, conflicting incentives between physicians and hospitals are often cited as a major obstacle to effective collaboration and threatens the long-standing assumption that physicians and hospitals share common interests [5,6]. Prior research has offered a number of important insights into alignment of the medical staff with the goals and objectives of the hospital. Three approaches can be identified. The first approach is rooted in the economic literature, building on the model of the homo economicus, in which alignment is realized by 'hard' financial means (economic integration). The second represents a more 'soft' sociological perspective, emphasizing the cooperative nature of their relationship (noneconomic integration). The third focuses on the clinical dimension of their relation, the coordination of patient care (clinical integration). In this paper we focus primarily on the first (economic integration) and second category (noneconomic integration). It has been argued that clinical integration is the apex of the three and is causally dependent on the development and successful execution of the other two [7,8]. As a result we argue that clinical integration is an outcome of alignment, defined as the degree to which physicians and hospitals share the same mission and vision, goals and objectives, and strategies, and work toward their accomplishment [9].

**Figure 1:**
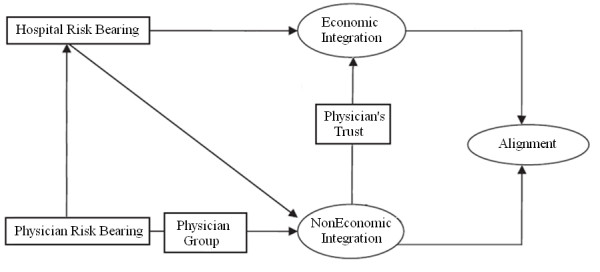
**An integrative model of physician hospital alignment**.

In this paper we focus on the importance of an effective, high quality relationship between hospitals and their medical staff resulting in increased alignment between both. Up to now there has not been an attempt to integrate the sociological perspective with the economic insights. We attempt to address this knowledge gap by developing a conceptual framework resulting in a practical and holistic understanding of physician hospital alignment. The model as depicted in figure 1 proposes relationships between important antecedents and physician hospital integration. First, provider financial risk bearing is identified as the main reason for increased integration between hospitals and their medical staff. However, because physicians mostly operate in a group setting, physician's individual financial risk bearing is pooled at the group level. Consequently it is important to incorporate physician financial risk bearing at the individual - and the group level. Furthermore, we argue that both integration strategies should be seen as complementary, rather than isolated strategies as there is an anticipated causal effect between both. As such, this paper proceeds previous work and deals with Granovetter's embeddedness paradigm that an inquiry focusing solely on economic or social aspects is not an accurate view [10]. Accordingly, next to risk-antecedent, representing the economic perspective, the sociological perspective - represented by trust - has been included when investigating physician-hospital alignment. More specifically, we argue that by building trust through non-economic integration strategies, increased financial risk sharing between both can be realized.

However, it should be noted that when alignment is considered as a development process in a longitudinal sense, outcomes can cause feedback and have a recursive relationship with the integration strategies. Similarly, economic integration also influences hospital and physician risk bearing. Therefore, we note that this model is a partial model and cannot represent all possible antecedents and consequences of physician hospital integration.

## Discussion

### Theoretical background

Several theories have been developed that offer useful insights in the complex, interdependent relationship between hospitals and physicians. This paper draws on agency theory [11] and social exchange theory [12] to increase understanding of the mechanisms used to align their interests. The principal goal of agency theory is to determine the most efficient contract which is considered a highly relevant aspect of our research problem. Specifically, agency theory describes the dilemma present when a principal engages another party, the agent, to perform a service. The agent does not have exactly the same objectives or motivations as the principal and does not necessarily act in the best interest of the principal. The principal goal of agency theory is to determine the most efficient contract using a unique framework based on outcome uncertainty, the associated financial risk and information asymmetry. Consequently, the principles of agency theory provide a useful framework to study economic integration strategies [13,14]. Although agency theory can be described as one of the most influential and widely used theories to study problems of relationships with a cooperative structure, additional theories can help to capture the greater complexity and improves understanding [11]. More specifically, in case of physician hospital alignment, the importance of noneconomic integration strategies is difficult to capture with the agency theory framework. We argue that social exchange theory can be a very useful perspective for the study of these non-economic integration strategies. According to this theory organizational members tend to reciprocate beneficial treatment they receive with positive work-related behavior and tend to reciprocate detrimental treatment they receive with negative work-related behavior [12,15]. In this sense, a good underlying cooperative relationship with the medical staff leads to increased alignment.

### The need for alignment

Internationally, hospitals are confronted with continuous pressures to contain costs and simultaneously improve health care quality. As a consequence, the relationship between hospitals and their medical staff has changed significantly over the past several decades. Traditionally physicians have been relatively independent of hospitals and have used them as workshops in which they carry out their professional services. The Hospital Physician Relationship (HPR) was characterized by unique, symbiotic interdependence in which the two parties had compatible incentives to increase the volume of care using the latest technology, while maximizing the professional autonomy of the physician [16]. This professional autonomy was reinforced by the fragmented financing system, which ignored the interrelatedness of the actions of physicians and hospitals in the treatment of their patients. Physicians were paid on a fee-for-service basis and hospitals were paid on the basis of costs incurred [17]. However, the financial relationship between hospitals and physicians has changed. Not only have margins declined due to increased complexity, rising costs and more restrictive reimbursement schemes [18], providers are also confronted with increased financial accountability for the delivered care, introduced by methods of prospective payment and forms of managed competition [19,20]. Furthermore, recognition that the health care system suffers from serious gaps in quality (i.e. medical errors, unnecessary differences in practice patterns and unintended variation in outcomes) has stimulated a broad array of public-, and private-sector initiatives to improve performance [21]. Accreditation, public reporting of hospital quality and value based purchasing (i.e. pay for quality) have become the locus of debate and have emerged as widely advocated strategies [22]. As a result, hospitals and physicians are no longer insulated from the financial consequences of their decisions. Finally, next to the degree of provider financial risk installed by the base compensation scheme and regulatory framework the degree of risk assumed by the hospital also depends on the alignment of incentives with the medical staff [23]. Given the physician autonomy in medical decision making, the medical staff controls many patient care decisions that influence hospital costs and quality, and by extension hospital financial performance. Consequently the degree of risk assumed by the hospital also depends on the alignment of incentives with the medical staff. More specifically, in the situations where the hospital bears a certain degree of financial risk (e.g. per case payment) and the medical staff's financial responsibility for their actions remains obsolete or limited (e.g. fee-for-service) the hospital's risk is considerably increased.

### The medical group level

Previous research on physician incentives identified the size and compensation structure of the medical group as an important matter to the risk distribution problem inherent to health care delivery. The group level creates an important possibility to limit individual financial risk by pooling the risk within the group. This results in 'risk pools' which can be described as a number of physicians that are paid collectively and thus share financial risk for the cost of patient care [24]. As the individual physicians are sometimes paid on a different basis than the group, a risk adjustment can be made at the individual practitioner level. Therefore, risk assumption may operate at different levels in organizational settings, the first via a group effect and the latter at the individual physician level [25,26]. We argue that this group level has an important buffering effect in aligning financial incentives between the hospital and the medical staff. In a similar vein, the recent discussion in the US about the role of accountable care organizations in future health care delivery reflects our argument. This new type of organization is built around providers and differs from historical managed care organizations (primarily health maintenance organizations). Rather than holding insurers at full financial risk for the cost of care these organizations focus on provider financial risk bearing at the group level [27].

### Noneconomic Integration

Theoretically rooted in social exchange theory, noneconomic integration strategies aim at optimizing the working relationship between the hospital and the medical staff. Research focusing on these strategies suggests that more emphasis should be placed on the underlying cooperative aspects of their relationship instead of the contractual, economic ties [9]. Within previous research, a distinction can be drawn between administrative linkages related to shared decision making and operational linkages focusing on supporting physicians in practicing medicine. First, it has been argued that physician involvement in planning and decision making holds a great promise for aligning hospital and physician interests [28]. This form of noneconomic integration is believed to increase their fiduciary responsibility and exposure to tough decisions, both of which are likely to increase physician sensitivity to hospital performance and the creation of a more cooperative decision-making environment [29]. Second, aiming at the provision of value-added contributions to the physician(group), operational support can be a valuable instrument to increase alignment. It allows the physicians to operate more effectively and efficiently in a complex and changing healthcare environment in which they have to deal with a myriad of demands. These operational linkages create true interdependence by providing valued resources to the physician group, which results in increased organizational commitment from the physicians receiving these resources [30].

### Economic Integration

Building further on the agency framework, we now concentrate on shared risk and gains in order to realize alignment. Within previous research, the question how financial incentives affect physician decision-making has been frequently addressed and it is widely believed that the method of payment of physicians affects their clinical and professional behavior [31]. However, we argue that the analysis of financial incentives cannot be separated from the base compensation scheme by which the providers are paid. This base compensation scheme creates its own incentives, which the supplemental economic incentives reinforce or counteract to realize increased alignment [32]. Consequently, the effect and use of economic incentives varies according to this base compensation. Given the variance in base compensation, this makes a review and interpretation of the findings about the effect of economic integration strategies difficult. We respond to this challenge by incorporating the macro level into the model by the risk antecedent. Based on agency theory, we argue that the base compensation scheme results in a varying risk distribution to the hospital and physician, on which supplementary economic alignment can be realized by a financial agreement (e.g. gainsharing and physician ownership).

### Organizational Trust

Next to risk, organizational trust lies at the heart of the management field and is vital in examining the principal-professional exchange [33]. In case of the hospital physician relationship, it is considered to be a social antecedent and critical concern of both parties [9]. Trust can be described as the willingness to be vulnerable to actions of another party irrespective of the ability to monitor or control that other party, making the risk antecedent, the driving force behind our conceptual framework, an essential component of trust [34,35]. Following agency theory, economic integration strategies give the possibility to align the interests of the physician by means of a contract. However, physicians may see little value added form their economic ties to hospitals. They even may view such connections as burdensome, if not antithetical to the traditional values of autonomy and freedom of external control [36]. Therefore next to the assessment of the risk by weighing the likelihood of positive and negative outcomes that might occur, trust can be considered crucial in intensifying the economic ties with the medical staff. In our model, we conceptualize non-economic integration strategies as a complementary management approach, primarily rooted in social exchange theory. Trust has emerged as a central concept within this theory and it has consistently been found as an outcome of co-operative behavior [37]. Therefore, we argue that by including trust as an antecedent to alignment we increase significantly the explanatory power of our model.

## Summary

The purpose of this article was to rethink physician hospital alignment. It extends current research by developing a conceptual framework incorporating both economic and noneconomic alignment and the causal relationship between both. This conceptual framework synthesizes insights from the literature and provides a holistic understanding of the interdependent relationship between hospitals and their medical staff. In doing so, this study challenges scholars and practitioners to consider the complexity inherent to the alignment problem more holistically. Additionally it may provide guidance for future research from a variety of different disciplines.

Our discussion has shown that hospitals are charged with developing internal organizations where quality and cost effectiveness are at the center of their attention. Consequently the historical separation of administrative and clinical decision making is eliminated. Unfortunately, conflicting incentives between physicians and hospitals are often a major obstacle to effective collaboration and alignment of the medical staff with the hospital objectives and goals. In our paper we argue that noneconomic integration lies at the very basis of alignment. It contributes directly to alignment through the norm of reciprocity and indirectly by building trust with the medical staff, laying the foundation for alignment of financial incentives.

## Competing interests

The authors declare that they have no competing interests.

## Authors' contributions

J.T. has been in charge of the literature search and developing the main arguments presented in the article, has led initial drafting of the article and has written the final manuscript. P.G. and L.A. have made substantial contributions to developing the arguments in the article and provided critical edits to the manuscript. All authors read and approved the final manuscript.

## Pre-publication history

The pre-publication history for this paper can be accessed here:

http://www.biomedcentral.com/1472-6963/11/36/prepub
